# Does Beta-Blockade Reduce the Risk of Depression in Patients with Isolated Severe Extracranial Injuries?

**DOI:** 10.1007/s00268-017-3935-5

**Published:** 2017-03-06

**Authors:** Rebecka Ahl, Galinos Barmparas, Louis Riddez, Eric J. Ley, Göran Wallin, Olle Ljungqvist, Shahin Mohseni

**Affiliations:** 1 School of Medical Sciences, Orebro University, Fakultetsgatan 1, 702 81 Orebro, Sweden; 2 Division of Trauma and Emergency Surgery, Department of Surgery, Orebro University Hospital, 701 85 Orebro, Sweden; 3 Division of Acute Care Surgery and Surgical Critical Care, Department of Surgery, Cedars-Sinai Medical Center, 8700 Beverly Blvd, Suite 8215N, Los Angeles, CA 90048 USA; 4 Division of Trauma and Emergency Surgery, Department of Surgery, Karolinska University Hospital, 171 76 Solna, Stockholm, Sweden

## Abstract

**Background:**

Approximately half of trauma patients develop post-traumatic depression. It is suggested that beta-blockade impairs trauma memory recollection, reducing depressive symptoms. This study investigates the effect of early beta-blockade on depression following severe traumatic injuries in patients without significant brain injury.

**Methods:**

Patients were identified by retrospectively reviewing the trauma registry at an urban university hospital between 2007 and 2011. Severe extracranial injuries were defined as extracranial injuries with Abbreviated Injury Scale score ≥3, intracranial Abbreviated Injury Scale score <3 and an Injury Severity Score ≥16. In-hospital deaths and patients prescribed antidepressant therapy ≤1 year prior to admission were excluded. Patients were stratified into groups based on pre-admission beta-blocker status. The primary outcome was post-traumatic depression, defined as receiving antidepressants ≤1 year following trauma.

**Results:**

Five hundred and ninety-six patients met the inclusion criteria with 11.4% prescribed pre-admission beta-blockade. Patients receiving beta-blockers were significantly older (57 ± 18 vs. 42 ± 17 years, *p* < 0.001) with lower Glasgow Coma Scale score (12 ± 3 vs. 14 ± 2, *p* < 0.001). The beta-blocked cohort spent significantly longer in hospital (21 ± 20 vs. 15 ± 17 days, *p* < 0.01) and intensive care (4 ± 7 vs. 3 ± 5 days, *p* = 0.01). A forward logistic regression model was applied and predicted lack of beta-blockade to be associated with increased risk of depression (OR 2.7, 95% CI 1.1–7.2, *p* = 0.04). After adjusting for group differences, patients lacking beta-blockers demonstrated an increased risk of depression (AOR 3.3, 95% CI 1.2–8.6, *p* = 0.02).

**Conclusions:**

Pre-admission beta-blockade is associated with a significantly reduced risk of depression following severe traumatic injury. Further investigation is needed to determine the beneficial effects of beta-blockade in these instances.

## Introduction

Symptoms of depression and anxiety may occur in up to half of all patients following significant trauma, resulting in poorer outcomes in functional recovery and quality of life [1, 2]. Established depression is difficult to treat and a preventative measure is therefore in the interest of both patients and trauma physicians [3]. The mechanisms resulting in clinical depression are not established but intense emotion around the time of trauma is of importance for subsequent ‘memory recollection’ and thereby depression maintenance [4]. During memory retrieval of traumatic events, adrenergic activity is increased in humans and is manifested as increased heart rate and skin conduction. These physiologic findings are more significant in patients diagnosed with post-traumatic stress disorder (PTSD) [5]. Animal studies show that noradrenaline levels in the brainstem increase with trauma and amnesia is induced in rodents that are administered systemic propranolol shortly after memory recollection [6, 7]. In clinical studies, subjects who received metoprolol showed diminished physiologic stress responses when watching a traumatic event and had impaired memory recall [8]. Consequently, it has been hypothesized that β-adrenergic receptor activation plays an important role in the early stages of trauma-induced depression and indicates a potential therapeutic role for β-blockade (BB) in trauma patients. BB treatment administered peri- and post-trauma has been investigated in the context of PTSD and appears to reduce such symptoms [9–12], but studies are limited in cohort size and conflicting results have been demonstrated [13–15]. The effect of pre-injury BB on the development of clinical depression has not yet been studied, which is the aim of the current work.

## Materials and methods

Ethical approval for this retrospective cohort study was obtained from the Regional Review Board (Stockholm County) and the study adhered to the Declaration of Helsinki. The study cohort was identified by querying the trauma registry of Karolinska University Hospital, an urban university hospital, for patients admitted to the trauma unit with severe extracranial injuries (SECI) between January 1, 2007, and December 31, 2011. SECI were defined as any extracranial injury (head, neck, thorax, abdomen, extremity, spine) with Abbreviated Injury Scale (AIS) score ≥ 3, intracranial AIS < 3 and an overall injury burden set by the Injury Severity Score (ISS) ≥ 16. Individual patient charts were reviewed to characterize head injuries such that only those patients with a head AIS of ≥ 3 due to intracranial injury (International Classification of Diseases 10th Revision S06.1–S06.9) were excluded. All adult patients (≥18 years) were included. In-hospital deaths were excluded as were patients prescribed antidepressants up to 1 year prior to the studied admission. Additional ethical approval was obtained from the National Board of Health and Welfare (Socialstyrelsen) for access to the national drugs registry database. Information was retrieved for β-blocker therapy 12 months prior to admission and antidepressant (selective serotonin reuptake inhibitors, serotonin–norepinephrine reuptake inhibitors, tricyclic antidepressants and monoamine oxidase inhibitors) therapy within 12 months prior and following the hospital stay. Post-traumatic depression was ascribed to patients given antidepressant therapy within 1 year of discharge. Patient variables including age, gender, injury mechanism, AIS, ISS, International Statistical Classification of Diseases (ICD) 10th version for traumatic injuries, admission Glasgow Coma Scale (GCS) score, intensive care unit length of stay (ICU LOS) and hospital length of stay (HLOS) were obtained from the trauma registry. A retrospective review of electronic patient records was carried out for clinical information during the admission.

## Statistical analysis

Demographic and clinical information of the total study cohort and variable differences between the cohorts dependent upon pre-admission BB were analyzed using Chi-squared test for categorical data and Student’s *t* test, or Mann–Whitney U test, when appropriate, for continuous data. For subsequent analysis, continuous variables were dichotomized based on cutoff points recognized as having clinical relevance in trauma outcomes: age ≥ 55 vs. < 55 years, ISS ≥ 25 vs. < 25, AIS ≥ 4 vs. < 4, GCS ≤ 8 vs. > 8. Bivariate analysis was performed based on these dichotomous variables. Variables with *p* ≤ 0.2 from the bivariate analysis were entered into a multivariable forward logistic regression model to isolate independent risk factors for depression. The study population was stratified by pre-admission BB exposure, and a multivariable logistic regression, adjusted for significant differences (*p* ≤ 0.05) between groups, was carried out to examine the effect of BB on depression. Analysis was performed using Statistical Package for the Social Sciences (SPSS Macintosh^®^) version 23.

## Results

A total of 774 patients with SECI were identified from the trauma registry and of those 596 met the inclusion criteria. The mean age was 44 ± 18 years, 82% were male, and 89% of injuries were caused by blunt trauma with a mean ISS of 23 ± 8. Pre-admission BB was prescribed in 11.4% (*n* = 68). Comparison of demographics between the β-blocked group (BB^(+)^) and the group not prescribed pre-admission β-blockade (BB^(−)^), demonstrated that BB^(+)^ patients were older (57 ± 18, vs 42 ± 17 years, *p* < 0.001) and had lower admission GCS score (12 ± 3 vs 14 ± 2, *p* < 0.001) (Table 1). Overall, depression occurred in 13.8% of patients and the reduction observed in those who received BB did not reach significance (8.8% vs 14.4%, *p* = 0.20). The BB group spent more days in intensive care (4 ± 7 vs. 3 ± 5 days, *p* = 0.01) and had longer hospital stays overall (21 ± 20 vs. 15 ± 17 days, *p* < 0.01) (Table 2). Patient variables were entered into a bivariate analysis. β-Blocker exposure, gender, ISS, AIS, GCS, abdominal injury, multiple injuries, surgery and length of ICU and overall hospital stay demonstrated a plausible association (*p* ≤ 0.2) with the development of post-trauma depression (Table 3). In order to determine independent risk factors for depression, the seven variables were subsequently entered into a multivariable forward logistic regression model. As depicted in Table 4, in addition to the lack of BB exposure (OR 2.7; 95% CI 1.1–7.2; *p* = 0.04), AIS ≥ 4, GCS ≤ 8 and surgery were identified as independent risk factors for developing depression. Following adjustment for significant differences between the cohorts, the relationship between lack of pre-admission BB and development of post-trauma depression was strengthened (AOR 3.3; 95% CI 1.2–8.6; *p* = 0.02) (Table 5).

**Table 1:** Demographic differences between patients who were and were not prescribed pre-admission β-blockers

Variable	Total *n* = 596	BB^(−)^ *n* = 528	BB^(+)^ *n* = 68	*p*
Male (%)	82	83	75	0.10
Blunt injury (%)	89	89	93	0.34
Age, mean ± SD	44 ± 18	42 ± 17	57 ± 18	<0.001
Age ≥ 55 years (%)	26	21	59	<0.01
ISS, mean ± SD	23 ± 8	23 ± 8	24 ± 8	0.26
ISS ≥ 25 (%)	33	33	32	0.95
AIS, mean ± SD	3.8 ± 0.7	3.8 ± 0.7	3.8 ± 0.8	0.61
Highest AIS ≥ 4 (%)	60	60	53	0.24
GCS, mean ± SD	14 ± 2	14 ± 2	12 ± 3	<0.001
GCS ≤ 8 (%)	9	5	35	<0.01
Head/spine injury (%)	20	20	18	0.69
With AIS ≥ 4 (%)	75	77	58	0.52
Abdominal injury (%)	23	23	22	0.90
With AIS ≥ 4 (%)	90	90	93	0.68
Thoracic injury (%)	32	32	34	0.76
With AIS ≥ 4 (%)	63	64	57	0.33
Extremity injury (%)	5	6	3	0.35
With AIS ≥ 4 (%)	34	33	50	0.16
Multiple injury (%)	20	20	24	0.48
with AIS ≥ 4 (%)	12	12	6	0.45

*BB* beta-blockers, *ISS* Injury Severity Score, *AIS* Abbreviated Injury Scale, *GCS* Glasgow Coma Scale

**Table 2:** Clinical outcome differences between patients who were and were not prescribed pre-admission β-blockers

Variable	Total *n* = 596	BB^(−)^ *n* = 528	BB^(+)^ *n* = 68	*p*
Surgery (%)	57	56	60	0.51
ICU LOS, mean days ± SD	3 ± 5	3 ± 5	4 ± 7	0.01
HLOS, mean days ± SD	16 ± 17	15 ± 17	21 ± 20	<0.01
Depression (%)	13.8	14.4	8.8	0.20

*BB* beta-blockers, *ICU LOS* intensive care unit length of stay, *HLOS* hospital length of stay

**Table 3:** Bivariate relationship between variables and the development of depression with *p* ≤ 0.2 included in subsequent multivariable analysis

Categorical variable		Depression, *n* (%)	OR (95% CI)	*p*
β-Blocker	No	76/528 (14.4)	1.7 (0.7–4.1)	0.20
	Yes	6/68 (8.8)		
Male	Yes	63/490 (12.9)	1.5 (0.8–2.6)	0.17
	No	19/106 (17.9)		
Blunt injury	Yes	71/532 (13.3)	0.7 (0.4–1.5)	0.40
	No	11/64 (17.2)		
Age ≥ 55 years	Yes	22/153 (14.4)	1.1 (0.6–1.8)	0.80
	No	60/443 (13.5)		
ISS ≥ 25	Yes	37/195 (19.0)	1.9 (1.2–3.0)	0.01
	No	45/401 (11.2)		
Highest AIS ≥ 4	Yes	61/335 (17.2)	2.2 (1.3–3.7)	<0.01
	No	21/241 (8.7)		
GCS ≤ 8	Yes	14/52 (26.9)	2.6 (1.3–5.0)	<0.01
	No	68/544 (12.5)		
Head/spinal injury	Yes	16/116 (13.8)	1.0 (0.5–1.8)	0.99
	No	66/480 (13.8)		
Thoracic injury	Yes	24/192 (12.5)	0.9 (0.5–1.4)	0.54
	No	58/404 (14.4)		
Abdominal injury	Yes	24/135 (17.8)	1.5 (0.9–2.5)	0.12
	No	58/461 (12.6)		
Extremity injury	Yes	6/32 (18.8)	1.5 (0.6–3.7)	0.40
	No	76/564 (13.5)		
Multiple injuries	Yes	12/121 (9.9)	0.6 (0.3–1.2)	0.17
	No	70/475 (14.7)		
Surgery	Yes	63/337 (18.7)	2.9 (1.7–5.0)	<0.01
	No	19/259 (7.3)		

Continuous variable	No depression	Depression	Mean difference	*P*
HLOS ± SD	15 ± 16	23 ± 20	7.9 (3.9–11.8)	<0.001
ICU LOS ± SD	3 ± 5	4 ± 5	0.9 (−0.4–2.2)	<0.01

*ISS* Injury Severity Score, *AIS* Abbreviated Injury Scale, *GCS* Glasgow Coma Scale, *ICU LOS* intensive care unit length of stay, *HLOS* hospital length of stay

**Table 4:** Independent predictors for depression by multivariable logistic regression

Step	Variable	OR (95% CI)	*p*
1	Surgery	2.9 (1.7–5.0)	<0.01
2	Highest AIS ≥ 4	2.1 (1.3–3.6)	<0.01
3	GCS ≤ 8	2.1 (1.1–4.2)	0.03
4	No pre-admission β-blocker	2.7 (1.1–7.2)	0.04

The following variables were included in the multivariable analysis: beta-blocker, gender, ISS, GCS, AIS, abdominal injury, multiple injuries, surgery, hospital and ICU length of stay

*AIS* Abbreviated Injury Scale, *GCS* Glasgow Coma Scale

**Table 5:** Odds ratio for depression after adjustment for differences between the cohorts

	Depression, *n* (%)	AOR (95% CI)	*p*
No pre-admission β-blocker	76/528 (14.4)	3.3 (1.2–8.6)	0.02
Pre-admission β-blocker	6/68 (8.8)		

The following variables were adjusted for in the regression analysis: beta-blocker, age ≥ 55 years, GCS ≤ 8, hospital and ICU length of stay

A total of 47 patients had a minor intracranial injury (AIS ≤ 2) with concomitant severe (AIS ≥ 3) extracranial injuries. Of these, 11.1% (*n* = 1) of patients on beta-blockade developed depression compared to 18.4% (*n* = 7) in the non-β-blocked cohort with a *p* value of 0.60. A separate multi-regression analysis excluding these patients and adjusting for differences between the BB^(+)^ and BB^(−)^ cohorts still resulted in a three times greater risk of depression in the BB^(−)^ patients (adjusted OR 3.4, 95% CI 1.3–9.0, *p* = 0.02).

## Discussion

Symptoms of anxiety and depression are common in patients following severe trauma [1, 16]. It has been hypothesized that the catecholamine surge occurring at the time of trauma may be an underlying factor facilitating the formation of strong emotional memories that are easily maintained. Ruminating on memories is a significant contributory factor in the development and maintenance of clinical depression [17, 18]. BB inhibits adrenergic receptor activity and might inhibit the consolidation of such memories [7]. This inhibition, in turn, could blunt the emotional effect of traumatic events and prevent post-injury depression [8]. Interestingly, the use of pindolol together with serotonergic antidepressants has previously demonstrated a synergistic effect on negative moods [19]. Regular BB intake also appears to have a protective role in reducing emotional distress and depressive thoughts in the context of cancer diagnoses as well as following percutaneous coronary intervention in ischemic heart disease [20, 21]. These studies support the current findings that BB may play a role in the prevention of post-traumatic depression.

The current study shows that 13.8% of the total cohort commenced antidepressant therapy within 12 months of sustaining severe extracranial injury. The incidence of clinical depression was lower in the group exposed to BB prior to the traumatic event (8.8%) compared to those who were not (14.4%). The average prevalence of patients prescribed antidepressant therapy in the county of Stockholm during the studied period (2007–2011) varied between 9.1 and 9.4% yearly [22]. This suggests that trauma patients have an increased risk of developing depression post-trauma. Furthermore, our findings suggest that depression is a significant comorbidity in the trauma population and provides data that BB might be effective at prevention. The mechanism of post-trauma depression is likely to be multifactorial, and this study identifies four independent risk factors. Patients who lacked β-blockade demonstrated a threefold increase in becoming depressed compared to those who were on the medication. Further, low GCS on admission, more severe injury and requiring surgery were associated with increased risk of post-traumatic depression.

Interestingly, despite the reduction in patient depression associated with pre-admission BB, the unadjusted reduction in the incidence of depression between groups (BB^(+)^ vs. BB^(−)^) did not achieve statistical significance (*p* = 0.20). However, the unadjusted analysis does not account for confounding factors such as the fact that patients who had been prescribed β-blockers had significantly longer ICU stays and were older; both factors associated with depressive syndromes [23, 24]. For example, trauma patients admitted to the ICU have been estimated to have a four times greater risk of developing moderate depression, anxiety or stress symptoms 6 months post-injury [1]. Additionally, the observed protective effect of BB in this study increased when adjustment was made for significant group differences in the logistic regression model, strengthening the observed impact of BB on the risk of depression.

While our findings provide support for a possible BB effect on post-trauma depression, the current retrospective design does not establish a direct causal link. Further investigation is required into the mechanisms behind this effect, and a prospective design examining whether early β-blockade during the admission itself results in the same outcome would be of value. Additionally, the national drugs registry neither keeps record of alternative therapies for depression such as electroconvulsive or talking/behavioral therapies, nor does it register the medical indication for prescriptions. Except for the exclusion of patients’ prescribed pre-admission antidepressants, the study design is unable to control for pre-injury comorbidities such as anxiety and substance abuse, which may entail a certain overlap with depressive traits. Finally, the study does not control for the type of β-blocker (i.e., selective vs. non-selective and lipophilic vs. hydrophilic) although over 70% of patients were prescribed metoprolol. Due to this overwhelming majority, all patients with prescribed β-blockers were treated identically and no subgroup analyses were performed. However, since lipophilic β-blockers are able to cross the blood–brain barrier, this subgroup may be of greater relevance in the context of depression prevention. A subsequent study subgrouping different types of BB might therefore be of value.

## Conclusion

The exposure to pre-admission β-blockers significantly reduces the risk of developing depression by a factor of three up to 1 year following severe traumatic extracranial injury. Further investigation is needed to explore whether β-blockade in these instances may have a prophylactic, or possibly therapeutic, role.

## References

[1] Wiseman TA, Curtis K, Lam M (2015). Incidence of depression, anxiety and stress following traumatic injury: a longitudinal study. Scand J Trauma Resusc Emerg Med.

[2] Jorge RE, Robinson RG, Tateno A (2015). Major depression following traumatic brain injury. Arch Gen Psychiatry.

[3] Fava M (2003). Diagnosis and definition of treatment-resistant depression (1992). Biol Psychiatry.

[4] Christianson SA (1992). Handbook of emotion and memory: current research and theory.

[5] Brunet A, Orr SP, Tremblay J (2008). Effect of post-retrieval propranolol on psychophysiologic responding during subsequent script-driven traumatic imagery in post-traumatic stress disorder. J Psychiatr Res.

[6] Terziog B (2013). Increased noradrenaline levels in the rostral pons can be reversed by M1 antagonist in a rat model of post-traumatic stress disorder. Neurochem Res.

[7] Przybyslawski J, Roullet P, Sara SJ (1999). Attenuation of emotional and nonemotional memories after their reactivation: role of NA-adrenergic receptors. J Neurosci.

[8] O’Carroll RE, Drysdale E, Cahill L (1999). Stimulation of the noradrenergic system enhances and blockade reduces memory for emotional material in man. Psychol Med.

[9] Pitman RK, Sanders KM, Zusman RM (2002). Pilot study of secondary prevention of posttraumatic stress disorder with propranolol. Biol Psychiatry.

[10] Hoge EA, Worthington JJ, Nagurney JT (2012). Effect of acute posttrauma propranolol on PTSD outcome and physiological responses during script-driven imagery. CNS Neurosci Ther.

[11] Poundja J, Sanche S, Tremblay J (2012). Trauma reactivation under the influence of propranolol: an examination of clinical predictors. Eur J Psychotraumatol.

[12] Brunet A, Thomas É, Saumier D (2014). Durably low physiological responding during subsequent script-driven traumatic imagery. Can J Psychiatry.

[13] Spring JD, Wood NE, Mueller-pfeiffer C (2015). Prereactivation propranolol fails to reduce skin conductance reactivity to prepared fear-conditioned stimuli. Psychophysiology.

[14] Stein MB, Kerridge C, Dimsdale JE (2007). Pharmacotherapy to prevent PTSD: results from a randomized controlled proof-of-concept trial in physically injured patients. J Traum Stress.

[15] Wood NE, Rosasco ML, Suris AM (2015). Pharmacological blockade of memory reconsolidation in posttraumatic stress disorder: three negative psychophysiological studies. Psychiatry Res.

[16] Kessler RC, Sonnega A, Bromet E (1995). Posttraumatic stress disorder in the national comorbidity survey. Arch Gen Psychiatry.

[17] Nolen-Hoeksema S, Wisco BE, Lyubomirsky S (2008). Re-thinking rumination. Perspect Psychol Sci.

[18] Cooney RE, Joormann J, Eugéne F (2010). Neural correlates of rumination in depression. Cogn Affect Behav Neurosci.

[19] Whale R, Terao T, Cowen P (2010). Pindolol augmentation of serotonin reuptake inhibitors for the treatment of depressive disorder: a systematic review. J Psychopharmacol.

[20] Lindgren ME, Fagundes CP, Alfano CM (2013). Beta-blockers may reduce intrusive thoughts in newly diagnosed cancer patients. Psycho-oncology.

[21] Battes LC, Pedersen SS, Oemrawsingh RM (2012). Beta blocker therapy is associated with reduced depressive symptoms 12 months post percutaneous coronary intervention. J Affect Disord.

[22] The National Board of Health and Welfare’s statistical database for prescribed drugs. http://www.socialstyrelsen.se/statistik/statistikdatabas/lakemedel. Accessed 23 Oct 2016

[23] Davydow D, Gifford J, Desai S (2009). Depression in general intensive care unit survivors: a systematic review. J Intensive Care Med.

[24] Huang CQ, Dong BR, Lu ZC (2010). Chronic diseases and risk for depression in old age: a meta-analysis of published literature. Ageing Res Rev.

